# Albumin-Bilirubin Grade as a Novel Predictor of the Development and Short-Term Survival of Post-Banding Ulcer Bleeding Following Endoscopic Variceal Ligation in Cirrhotic Patients

**DOI:** 10.3390/medicina58121836

**Published:** 2022-12-13

**Authors:** Chun-Wei Chen, Chia-Jung Kuo, Chao-Wei Lee, Tony Kuo, Cheng-Tang Chiu, Chun-Jung Lin, Siew-Na Lim, Chau-Ting Yeh, Wey-Ran Lin

**Affiliations:** 1 Department of Gastroenterology and Hepatology, Linkou Chang Gung Memorial Hospital, Taoyuan 333, Taiwan; 2 Division of General Surgery, Department of Surgery, Linkou Chang Gung Memorial Hospital, Taoyuan 333, Taiwan; 3 College of Medicine, Chang Gung University, Taoyuan 333, Taiwan; 4 Department of Neurology, Linkou Chang Gung Memorial Hospital, Taoyuan 333, Taiwan; 5 Liver Research Center, Linkou Chang Gung Memorial Hospital, Taoyuan 333, Taiwan

**Keywords:** endoscopic variceal ligation, post-banding ulcer bleeding, liver cirrhosis, portal hypertension, MELD score, ALBI score, gastric varices, post-EVL ulcer bleeding, esophageal varices bleeding, retrospective study

## Abstract

*Background and Objectives*: Endoscopic variceal ligation (EVL) is the primary and secondary treatment for acute esophageal variceal bleeding. Post-banding ulcer bleeding (PBUB) may lead to bleeding episodes following EVL, increasing mortality. The aim of this study was to evaluate the risk factors for PBUB and predict the 6-week mortality risk after PBUB. *Materials and Methods*: We retrospectively analyzed the data collected from cirrhotic patients with EVL from 2015 to 2017. The incidence of PBUB and the 6-week mortality rate were evaluated. Risk factors for PBUB and predictive factors for mortality after PBUB were analyzed. *Results*: A total of 713 patients were enrolled in this study. Among the studied subjects, the incidence of PBUB was 5.8% (N = 41). The 6-week mortality rate was 63.4% (26/41). In multivariate analysis, MELD score ≥20 (OR: 3.77, 95% CI: 1.94–7.33, *p* < 0.001), ALBI score of 3 (OR: 2.67, 95% CI: 1.34–5.3, *p* = 0.005) and the presence of gastric varices (OR: 2.1, 95% CI: 1.06–4.16, *p* = 0.03) were associated with the development of PBUB. Patients with ALBI grade 3 (OR: 4.8, 95% CI: 1.18–19.6, *p* = 0.029) and Child-Pugh scores B and C (OR: 16.67, 95% CI: 1.75–158.1, *p* = 0.014) were associated with 6-week mortality after PBUB. *Conclusions*: PBUB is a complication with low incidence but increased mortality following EVL. The ALBI grade is a useful score to predict not only the development of PBUB but also the 6-week mortality after PBUB.

## 1. Introduction

Gastroesophageal varices are a major complication of liver cirrhosis. This complication occurs in 40% of patients with compensated cirrhosis and up to 85% of patients with decompensated cirrhosis [1]. Without management, acute bleeding from varices occurs in approximately 12% of cases per year and leads to life-threatening outcomes in cirrhotic patients [2]. Endoscopic variceal ligation (EVL) is an effective treatment not only to prevent esophageal variceal bleeding but also for acute bleeding episodes. However, it is associated with several complications such as esophageal stricture formation and post-banding ulcer bleeding (PBUB) [3]. Generally, with ligation bands applied on esophageal varices by endoscopy, the blood supply to the ligated mucosa is interrupted, which can lead to coagulative tissue necrosis and thrombosis in the adjacent varices [4]. Within 1 week after ligation, the necrotic esophageal tissue can develop ulceration, which usually heals within 2 to 3 weeks [5]. If the bands slip before the varices are occluded by mature thrombosis, bleeding from post-banding ulcers may occur.

Although the reported incidence of PBUB is low (2.3–7.7%), the mortality rate following PBUB is high (19–28%) [6,7,8,9,10]. Nevertheless, there are no clinical guidelines for the treatment of this lethal complication. To date, the risk factors for PBUB are not well established. Several studies have revealed that poor liver function is associated with PBUB, which is reflected by a high platelet ratio index (APRI) score, high model for end-stage liver disease (MELD) score and Child-Pugh score C [8,9,11,12]. Moreover, albumin-bilirubin (ALBI) grade is another useful scoring system to evaluate liver function, especially for patients with hepatocellular carcinoma treatment. However, little is known about which scoring systems have a higher predictive ability for PBUB events and short-term outcomes after PBUB. The aim of this study was to assess the risk factors and common liver function scoring systems for PBUB events and short-term clinical outcomes of PBUB following EVL among patients with liver cirrhosis.

## 2. Materials and Methods

### 2.1. Study Patients and Data Collection

From January 2015 to October 2017, patients undergoing EVL prophylactically and emergently in the endoscopic therapeutic center of a tertiary medical center in northern Taiwan were retrospectively included. A diagnosis of liver cirrhosis was made on compatible imaging findings and laboratory and endoscopic findings. Patients with missing laboratory data were excluded from the study. Within 4 weeks after EVL, patients suffering from major upper gastrointestinal bleeding symptoms including hematemesis, bloody stool and tarry stool episodes with confirmation that the bleeding was associated with post-banding ulcer by upper endoscopy were categorized into the PBUB group. Demographic, clinical laboratory and endoscopic data were collected at the time of EVL. The severity of liver function was determined by the Child-Pugh, MELD, APRI, FIB-4 and ALBI scores. The APRI score was calculated as ([AST/upper normal limit]/platelet count [10^9^/L]) × 100 [13]. The FIB-4 score was calculated as (age (years) × AST [U/L])/(platelet count [10^9^/L] × √ALT [U/L]) [14]. The ALBI score was calculated as (log10 bilirubin × 0.66) + (albumin × −0.085), where bilirubin is in μmol/L and albumin is in g/L. ALBI was categorized into three grades: ALBI-1 (≤−2.60), ALBI-2 (>2.60 to −1.39) and ALBI-3 (>−1.39) [15]. This study was approved by the ethics committee of Chang Gung Memorial Hospital (reference no: 201701006B0) and was performed in line with the principles of the Declaration of Helsinki.

### 2.2. Statistical Analyses

Continuous variables were shown as the mean ± S.D. and compared using the Student’s *t*-test. Categorical variables were shown as absolute and relative frequencies and compared using the chi-square test. Univariate and multivariate logistic regressions were performed to determine the correlation of the risk factors with PBUB. Actuarial probabilities of survival were estimated by the Kaplan-Meier method and compared by the log-rank test. A *p* value of less than 0.05 was considered statistically significant. The statistical analyses were carried out using SPSS 22 (IBM Corp., Armonk, NY, USA) for Windows.

## 3. Results

### 3.1. Patient Demographics and Outcomes of the PBUB Group

A total of 2046 EVL procedures were performed during the study period and 713 patients without missing data were included. Of these 713 patients, 41 (5.7%) suffered from PBUB within 4 weeks after EVL. The baseline characteristics of the PBUB and non-PBUB groups are shown in Table 1. The PBUB group was significantly younger than the PBUB group (53.95 ± 11.53 vs. 58.35 ± 12.48, *p* = 0.028). Regarding laboratory findings, the prothrombin time, total bilirubin and alanine aminotransferase were significantly higher for the PBUB group than for the non-PBUB group, and albumin was significantly lower for the PBUB group than for the non-PBUB group. In addition, the ALBI and MELD scores were significantly higher for the PBUB group (−1.00 ± 0.74 vs. −1.42 ± 0.65, *p* < 0.001; 22.9 ± 9.37 vs. 17.5 ± 8.25, *p* < 0.001, respectively).

**Figure 1:**
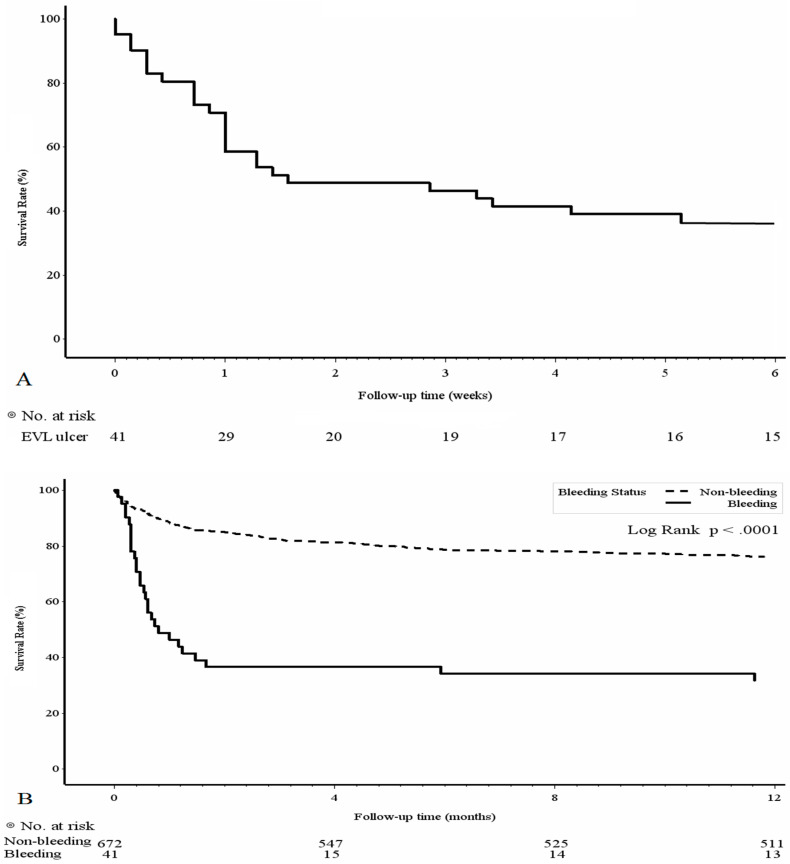
(**A**) Survival rate of PBUB group within 6 weeks; (**B**) Kaplan-Meier survival curves with a log-rank test between PBUB and non-PBUB groups within 1 year.

In the PBUB group, 29 (70.7%) patients were male and the time to PBUB was 8.41 ± 4.96 days. The bleeding-related 6-week mortality rate for the PBUB group was 63.4% (26/41) and the time to PBUB-related mortality was 9.15 ± 9.43 days. The 6-week survival curve of the PBUB group is shown in Figure 1A. The comparison of the survival rate within 1 year after EVL between the PBUB and non-PBUB groups is shown in Figure 1B.

### 3.2. Predictive Factors of PBUB

To evaluate the predictors of PBUB, univariate and multivariate analyses were conducted, and the results are shown in Table 2. In univariate analysis, prothrombin time (INR) >2.3, total bilirubin >3.0 mg/dL, sodium >150 mEq/L, MELD score ≥20 and ALBI grade 3 were associated with PBUB. For multivariate analysis, we selected the variables with a *p* value < 0.1 in the univariate analysis. The results showed that gastric varices and total bilirubin >3.0 mg/dL were associated with PBUB (OR: 2.1, 95% CI: 1.06–4.16, *p* = 0.03; OR: 3.57, 95% CI: 1.72–7.41, *p* = 0.001, respectively). As total bilirubin was a part of the MELD and ALBI scores, we included MELD score ≥20 and ALBI grade 3, respectively, in adjusted multivariate analysis with gastric varices. The results revealed that a MELD score ≥20 (OR: 3.77, 95% CI: 1.94–7.33, *p* < 0.001) and ALBI grade 3 (OR: 2.67, 95% CI: 1.34–5.3, *p* = 0.005) were associated with PBUB.

### 3.3. Mortality Risk Factors and Survival Analyses of the PBUB Group

**Figure 2:**
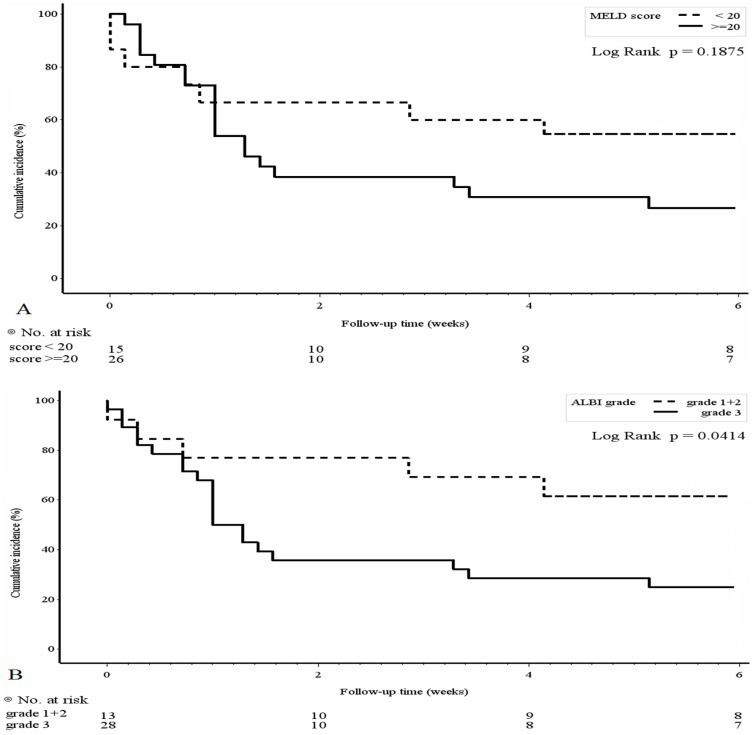

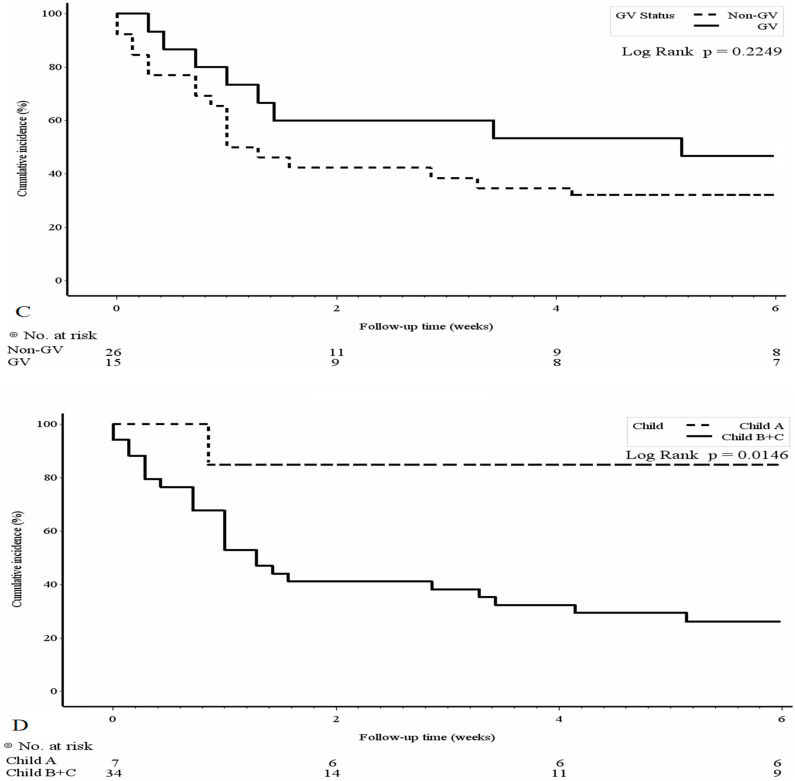
Kaplan-Meier estimates regarding the 6-week survival rate after post-banding ulcer bleeding stratified with (**A**) model for end-stage liver disease (MELD) score, (**B**) ALBI grade, (**C**) the presence of gastric varices and (**D**) Child-Pugh score.

To evaluate the clinical outcome of PBUB, we performed univariate analysis for 6-week mortality risk factors and the results are shown in Table 3. Child-Pugh scores B and C (OR: 16.67, 95% CI: 1.75–158.1, *p* = 0.014) and ALBI grade 3 (OR: 4.8, 95% CI: 1.18–19.6, *p* = 0.029) were associated with 6-week mortality. The Kaplan-Meier plot with log-rank analysis is shown in Figure 2 by MELD score, ALBI score, the presence of gastric varices and Child-Pugh score. Patients with ALBI grades 1 and 2 and Child-Pugh A showed significantly better outcomes within 6 weeks after PBUB.

## 4. Discussion

In this study, we demonstrated that patients with PBUB following EVL had a high mortality rate within 6 weeks, and patients without PBUB bleeding after EVL had significantly better 1-year survival outcomes than those with PBUB. Moreover, a MELD score ≥20, ALBI grade 3 and gastric varices were associated with the development of PBUB. Patients with ALBI grade 3 and Child-Pugh scores B and C were associated with 6-week mortality after PBUB. The ALBI grading score predicted not only the development of PBUB but also 6-week mortality after PBUB.

Our results echoed those from previous studies showing that the incidence of PBUB is low, but the risk of a lethal clinical outcome is high. The reported incidence of PBUB is between 2.3% and 7.7%, and the mortality rate following PBUB is approximately 20% to 30% [6,7,8,9,10]. Although the incidence of PBUB in our study is similar to that reported in previous studies, the 6-week mortality rate in our study was 63.4%, which was much higher than that reported in previous studies. In addition, our results also demonstrate a higher 1-year mortality rate for the PBUB group than for the non-PBUB group. Otherwise stated, PBUB caused not only short-term high mortality but also long-term high mortality among patients after band ligation. A poor liver function test led to poor outcomes after PBUB. The possible explanation for the high mortality rate in our study is that more than 80% of patients in the PBUB group had Child-Pugh scores B and C and the mean MELD score was 22.9, which was much higher than that reported in a previous study [9]. An increased MELD score was also associated with the risk of mortality among patients with acute variceal bleeding [16,17].

Our study demonstrates that ALBI grade 3 was a risk factor for PBUB (OR: 2.67, 95% CI: 1.34–5.3). Moreover, the 6-week mortality rate was significantly higher in patients with ALBI grade 3 than in those with ALBI grades 1 and 2. The ALBI grading system was originally developed in 2014 to assess liver dysfunction in patients with hepatocellular carcinoma (HCC) [15]. Several studies have suggested that ALBI grading is appropriate not only for the prognosis of HCC patients but also for patients with non-HCC liver disease [18,19,20]. Additionally, the ALBI grading system is applied to obtain an overall prognosis of post-HCC therapy, including surgical hepatectomy, radiotherapy, radiofrequency ablation, transarterial chemoembolization and systemic treatment [21]. One study in Japan suggested that increasing ALBI grading is associated with non-malignant mortality in cirrhotic patients [22]. ALBI grading has good clinical application to the nature and post-treatment outcome of liver disease. Furthermore, this is the first study to demonstrate that ALBI grade is associated not only with the development of PBUB but also with the 6-week clinical outcome of patients with PBUB. Child-Pugh scores B and C were other risk factors of 6-week mortality after PBUB. In our study, the odds ratio of 6-week mortality after PBUB was 16.67 (95% CI: 1.75–158.1). Our findings suggest that liver reserve function significantly impacted patient survival after PBUB. However, there were five major parameters to assess the Child-Pugh score, and only two parameters were used to assess the ALBI grade. Otherwise stated, ALBI grade was a good and simple parameter to guide the prognosis after PBUB.

A MELD score ≥20 was another risk factor for PBUB development in our multivariate analysis. The MELD score was developed to predict the 3-month mortality risk in patients with cirrhosis and severe liver dysfunction and is also applied to determine priority for liver transplantation waiting lists [23]. It is reasonable that the MELD score can predict the mortality risk of patients with acute variceal bleeding [16,17] and has been suggested for predicting the development of PBUB; the results of our study are in line with this finding. However, the MELD score failed to predict 6-week mortality in patients with PBUB in our study.

The presence of gastric varices was found to be another risk factor for PBUB in our study. Increased portal pressure leads to portal hypertension, and portosystemic collateral vessels develop gradually to decompress portal hypertension. As vein blood pressure and blood volume increase, esophageal and gastric varices form. In addition, spontaneous bleeding will develop without medical intervention. EVL is a good prophylactic endoscopic method to prevent excessive bleeding from esophageal varices. After EVL, although the esophageal varices were ligated, the portal pressure and portal blood flow did not decrease. If both gastric and esophageal varices are present, then portal blood flow is greater than when only esophageal varices are present. Thus, the increased blood volume may result in PBUB before the ligated mucosa heals. This may explain why gastric varices are a risk factor for PBUB.

We also acknowledge that there are several limitations to our study. First, this was a retrospective study conducted at one medical center. However, more than 700 patients were included, which is the second largest case number analyzing the risk for PBUB. In addition, more clinical variables were included that are more practical in the real world. Second, we did not analyze the influence of post-EVL medication, such as the usage of proton pump inhibitors and nonselective beta-blockers (NSBBs). Most of our patients were not given proton pump inhibitors as this medication is not recommended after EVL in the guidelines [1,24]. NSBBs are the medical treatment for the primary and secondary prevention of esophageal varices bleeding and are usually prescribed to stable cirrhotic patients. In our cohort, more than 85% of EVL was performed in acute bleeding conditions and the role of NSBBs was limited. Therefore, we did not analyze the effect of NSBBs. One study in Germany suggested that NSBBs do not lower the risk of PBUB after prophylactic EVL [8]. Third, some risk factors associated with PBUB were not analyzed in this study such as detachment of ligation bands and reflux esophagitis [9,10]. Both factors were difficult to access in acute bleeding conditions. In our cohort, more than 85% of EVL was performed emergently and post-EVL endoscopic evaluation was only performed in bleeding conditions. In our follow-up endoscopy, no residual bands were detected.

## 5. Conclusions

PBUB has a low incidence but a high risk of mortality after EVL. PBUB is associated with a MELD score ≥20, ALBI grade 3 and the presence of gastric varices. ALBI grade 3 and Child-Pugh scores B and C predict 6-week mortality after PBUB. In our study, a high ALBI score not only indicated an increased risk of developing PBUB but was also indicative of mortality risk in patients with PBUB.

## Figures and Tables

**Table 1:** Baseline demographic and clinical characteristics of the patients in PBUB and non-PBUB groups.

	PBUB, N = 41 (5.8%)	Non-PBUB, N = 672 (94.2%)	*p* Value
Sex, male	29 (70.7%)	480 (71.4%)	0.924
Age, years (Mean ± S.D.)	53.95 ± 11.53	58.35 ± 12.48	0.028
Cirrhosis etiology			0.348
HBV	11 (26.8%)	160 (23.8%)	
HCV	13 (31.7%)	208 (31.0%)	
Alcoholism	1 (2.4%)	110 (16.4%)	
HBV + alcoholism	9 (22.0%)	91 (13.5%)	
HCV + alcoholism	2 (4.9%)	29 (4.3%)	
HBV + HCV + alcoholism	0	8 (1.2%)	
Others	3 (7.3%)	45 (6.7%)	
Child-Pugh score			0.16
A	7 (17.1%)	181 (26.9%)	
B + C	34 (82.9%)	491 (73.1%)	
First EVL session	34 (82.9%)	466 (69.3%)	0.065
Gastric varices	15 (36.6%)	162 (24.1%)	0.073
Varices in endoscopy			0.535
F1	4 (9.8%)	47 (7.0%)	
F2	21 (51.2%)	401 (59.7%)	
F3	16 (39.0%)	224 (33.3%)	
Prophylactic	3 (7.3%)	94 (14.0%)	0.226
emergency	38 (92.7%)	578 (82.0%)	
Hepatocellular carcinoma	20 (48.8%)	284 (42.3%)	0.413
Portal vein thrombosis	5 (12.2%)	59 (8.8%)	0.458
Ascites	26 (63.4%)	400 (59.5%)	0.622
Hepatic encephalopathy	9 (22.0%)	138 (20.5%)	0.828
WBC (1000 u/L)	9.26 ± 6.20	7.98 ± 5.82	0.176
Hemoglobin (g/dL)	9.13 ± 1.90	9.26 ± 2.02	0.692
Platelet (1000 u/L)	105.93 ± 75.36	98.31 ± 65.85	0.476
P.T/INR	1.81 ± 0.56	1.56 ± 0.56	0.007
Creatinine (mg/dL)	1.60 ± 1.89	1.36 ± 1050	0.346
Total. Bilirubin (mg/dL)	9.57 ± 10.4	4.1 ± 6.47	<0.001
AST (U/L)	268.3 ± 732	150.4 ± 415.2	0.096
ALT (U/L)	117.9 ± 354.1	68.5 ± 126.6	0.04
Na (mEq/L)	138.1 ± 8.1	137.0 ± 5.8	0.249
K (mEq/L)	4.16 ± 0.85	3.98 ± 0.81	0.169
Albumin (g/dL)	2.67 ± 0.62	2.89 ± 0.57	0.018
APRI (mean ± S.D.)	7.68 ± 19.98	5.14 ± 14.58	0.29
FIB-4	15.39 ± 18.66	12.37 ± 17.16	0.277
ALBI	−1.00 ± 0.74	−1.42 ± 0.65	<0.001
MELD	22.9 ± 9.37	17.5 ± 8.25	<0.001

WBC: white blood count, HBV: hepatitis B virus, HCV: hepatitis C virus, EVL: endoscopic varices ligation; HE: hepatic encephalopathy; P.T: prothrombin time; INR: international normalized ratio; ALT: alanine aminotransferase, AST: aspartate aminotransferase, FIB-4: fibrosis 4, MELD: model for end-stage liver disease.

**Table 2:** Logistic regression analysis for delayed PBUB predictors.

	Univariate OR	95% CI	*p* Value	Multivariate OR	95% CI	*p* Value
Male	0.967	0.48–1.93	0.92			
Age (>50 y/o)	0.59	0.31–1.13	0.11	0.75	0.38–1.49	0.41
Alcoholism	1.54	0.82–2.9	0.17			
Child-Pugh score (B + C)	1.79	0.78–4.11	0.17			
Gastric varices	1.81	0.93–3.51	0.07	2.1	1.06–4.16	0.03
EV (F2 + F3)	0.69	0.23–2.03	0.5			
HCC	1.31	0.69–2.44	0.41			
PVT	1.44	0.54–3.81	0.46			
HE	1.08	0.5–2.33	0.82			
Ascites	1.18	0.61–2.26	0.62			
Emergent EVL	2.06	0.62–6.8	0.23			
First EVL	2.14	0.93–4.92	0.07	1.84	0.79–4.31	0.16
Hemoglobin (>8.0 g/dL)	0.91	0.45–1.82	0.79			
Thrombocytopenia (<150 K/uL)	0.76	0.34–1.7	0.5			
P.T (INR > 2.3)	2.78	1.10–7.01	0.03	1.2	0.44–3.35	0.69
Creatinine (>2.0 mg/dL)	1.73	0.80–3.75	0.16			
Total bilirubin (>3.0 mg/dL)	4.2	2.18–8.12	<0.001	3.57	1.72–7.41	0.001
AST (>200 U/L)	1.21	0.49–2.98	0.66			
ALT(>200 U/L)	0.76	0.18–3.29	0.72			
Na (>150 mEq/L)	3.71	1.02–13.46	0.04	2.56	0.67–9.78	0.17
K (>5.0 mEq/L)	0.93	0.322–2.68	0.89			
Albumin (>2.8 g/dL)	0.56	0.29–1.06	0.07	0.85	0.42–1.71	0.66
FIB-4	1.00	0.99–1.02	0.28			
MELD (≥20)	3.49	1.81–6.72	<0.001	3.77	1.94–7.33	<0.001 *
ALBI(grade3)	2.40	1.22–4.71	0.011	2.67	1.34–5.30	0.005 **

CI: confidence interval; OR: odds ratio; HCC: hepatocellular carcinoma; PVT: portal vein thrombosis; EVL: endoscopic varices ligation; HE: Hepatic encephalopathy; PT: prothrombin time; INR: international normalized ratio; ALT: alanine aminotransferase, AST: aspartate aminotransferase, MELD: model for end-stage liver disease. * Adjusted multivariate analysis with gastric varices alone. ** Adjusted multivariate analysis with gastric varices alone.

**Table 3:** Logistic regression analysis for PBUB mortality predictors.

	Univariate OR	95% CI	*p* Value
Child-Pugh A	1		
Child-Pugh B	33.0	2.45–443.6	0.008
Child-Pugh C	12.0	1.20–120	0.034
Child-Pugh score	16.67	1.75–158.1	0.014
(B + C)			
Gastric varices	1.97	0.53–7.31	0.31
MELD (≥20)	3.10	0.82–11.78	0.096
ALBI (grade3)	4.8	1.18–19.6	0.029

CI: confidence interval; OR: odds ratio; MELD: model for end-stage liver disease.

## Data Availability

The data that support the findings of this study are available from the corresponding author upon reasonable request.
